# Biomarkers of acute appendicitis: systematic review and cost–benefit trade-off analysis

**DOI:** 10.1007/s00464-016-5109-1

**Published:** 2016-08-05

**Authors:** Amish Acharya, Sheraz R. Markar, Melody Ni, George B. Hanna

**Affiliations:** Division of Surgery, Department of Surgery and Cancer, Imperial College London, 10th Floor QEQM Building, St Mary’s Hospital, South Wharf Road, London, W2 1NY UK

**Keywords:** Acute appendicitis, Biomarkers, Cost–benefit trade-off

## Abstract

**Background:**

Acute appendicitis is the most common surgical emergency and can represent a challenging diagnosis, with a negative appendectomy rate as high as 20 %. This review aimed to evaluate the clinical utility of individual biomarkers in the diagnosis of appendicitis and appraise the quality of these studies.

**Methods:**

A systematic review of the literature between January 2000 and September 2015 using of PubMed, OvidMedline, EMBASE and Google Scholar was conducted. Studies in which the diagnostic accuracy, statistical heterogeneity and predictive ability for severity of several biomarkers could be elicited were included. Information regarding costs and process times was retrieved from the regional laboratory. European surgeons blinded to these reviews were independently asked to rank which characteristics of biomarkers were most important in acute appendicitis to inform a cost–benefit trade-off. Sensitivity testing and the QUADAS-2 tool were used to assess the robustness of the analysis and study quality, respectively.

**Results:**

Sixty-two studies met the inclusion criteria and were assessed. Traditional biomarkers (such as white cell count) were found to have a moderate diagnostic accuracy (0.75) but lower costs in the diagnosis of acute appendicitis. Conversely, novel markers (pro-calcitonin, IL 6 and urinary 5-HIAA) were found to have high process-related costs including analytical times, but improved diagnostic accuracy. QUADAS-2 analysis revealed significant potential biases in the literature.

**Conclusion:**

When assessing biomarkers, an appreciation of the trade-offs between the costs and benefits of individual biomarkers is needed. Further studies should seek to investigate new biomarkers and address concerns over bias, in order to improve the diagnosis of acute appendicitis.

**Electronic supplementary material:**

The online version of this article (doi:10.1007/s00464-016-5109-1) contains supplementary material, which is available to authorized users.

Acute appendicitis is the most common surgical emergency, with an annual incidence in the USA of 9.38 per 100,000 [1]. Cases are characterized by an acute inflammatory process, but in approximately 16.5 % the appendix has perforated and become gangrenous or there is overt peritonitis, termed ‘complicated appendicitis’ [2]. Whilst in rare special circumstances management may differ, the mainstay of treatment for the majority of patients remains surgery either by an open or by laparoscopic approach. With 326,000 appendectomies performed in the USA during 2007, at an average estimated cost of $6242 [3], appendicitis represents a highly prevalent condition with significant expenditure associated with its treatment.

Despite the frequency of appendicitis, accurate diagnosis remains difficult. The National Surgical Research Collaborative in the UK has estimated that the negative appendectomy rate is as high as 20.6 % [2]. The use of ultrasound and computerized tomography (CT) has in some cases been shown to improve appendicitis diagnostic accuracy and reduce the number of negative appendectomies [7], with the latter shown to decrease rates to less than 10 % [4]. However, exposing patients to high levels of radiation is undesirable given the lifetime risk of cancer, along with the increase in costs associated with increased utilization of CT, representing negatives to this approach. Whilst this radiation dosage is avoided by using ultrasound scanning, the technique is operator dependent, and in as many as 55 % of cases the appendix fails to be visualized [5].

Several studies have previously examined a variety of biomarkers associated with appendicitis to more appropriately assign risk and allocate further diagnostic investigation. These have the potential of providing noninvasive objective criteria to aid clinicians in the diagnosis of appendicitis and in some cases predict the severity of the condition, with no adverse effects upon the patient. In several studies, biomarkers have been shown to have potentially good diagnostic accuracy and reliability, but with variable financial and timing implications. The latter significantly limits the clinical effectiveness of a biomarker in the emergency setting. The ‘ideal’ diagnostic biomarker would therefore maximize clinical utility and minimize procedural cost including analytical time. The aim of this study was to evaluate specific characteristics of biomarkers that surgeons’ value and to critically assess the cost–benefit of both traditional and novel biomarkers in the diagnosis of acute appendicitis from published literature.

## Materials and methods

### Literature search strategy

A literature search of PubMed, OvidMedline, EMBASE and Google Scholar electronic databases was conducted from January 1, 2000, up to and including September 1, 2015, for studies regarding the use of urine or serum biomarkers in the diagnosis of appendicitis or the prediction of complicated appendicitis. Search terms used included: *appendicitis, serum, blood, urine, biomarkers, diagnosis, diagnostics, perforation, complicated* and *severity* in various combinations, as well as the name of specific biomarkers previously identified.

Research titles were then screened for suitability, and full-text copies were retrieved. Further potentially appropriate papers were highlighted by assessing the reference lists and citations of the articles being screened. All studies that investigated the diagnostic ability of a single or multiple biomarkers that could be tested in the urine or blood of patients were included. Exclusion criteria involved studies with no available English translation, no full-text edition available, and those assessing the predictive ability of biomarkers for severity in which no diagnostic accuracy could be calculated.

Of those studies meeting inclusion criteria, the year of publication, population demographics, the number of patients enrolled and the stated specificity and sensitivity of the biomarker for diagnosis and severity were extracted. For studies that did not explicitly state the sensitivity and specificity of the biomarker, provided sufficient data were available, these were independently calculated.

### Literature standard

The QUADAS-2 tool was used to appraise the standard of the literature. It was implemented, as it has been previously described, to assess the quality and risk of bias of the included studies [6]. The tool involves four domains: patient selection, index test, reference standard and the flow of subjects through the study. Prompting questions are used to allow the reviewer to assess whether there is a risk of bias with respect to each of the four domains. It also allows the reviewer to gauge the applicability of the study to the review with respect to the first three domains. In this review, the reference standard is the histological examination of the appendix.

### Biomarker survey

General surgeon members of the European Association of Endoscopic Surgery (EAES) were asked to complete an anonymous survey regarding their opinions on the most desirable characteristics the ideal diagnostic biomarker of acute appendicitis would possess (Table 1). The surgeons were asked to rank each characteristic in the order of importance, including diagnostic benefits (high sensitivity, high specificity, reproducibility and predictive ability of perforation), process-related financial costs, time for result, ease of testing and patient acceptability. The average rank for each of the attributes, e.g., sensitivity, was then calculated, to identify which were the most desired characteristics. These ranks were used to inform the weightings for the cost–benefit trade-off, with greater importance placed upon higher ranked attributes.

**Table 1:** Definitions of the characteristics of biomarkers the consultants were asked to rank

Definitions	Outcome utilized
Sensitivity	Result of pooled sensitivity for diagnosis of acute appendicitis
Specificity	Results of pooled specificity for diagnosis of acute appendicitis
Predictive of perforation	Area under the curve of summary ROC for diagnosis of perforated appendicitis
Cost	Cost of investigation from Imperial College NHS Trust
East of testing	Level of invasiveness of testing
Acceptability	The impression of patient acceptability
Time for result	Time from sample being taken to result being available for clinician interpretation as described by Imperial College NHS Trust
Reproducibility	*I* ^2^ statistic for heterogeneity: increasing value indicates LESS consistency

### Statistical methodology

For each of the assessments of acute appendicitis and severity of appendicitis (perforation), paired sensitivity and specificity were calculated for diagnosis or severity, as appropriate, from each eligible study. A bivariate model for meta-analysis of statistical accuracy provides more accurate results than fixed-effect modeling. Following the validated methodology of Harbord et al. [7], bivariate meta-analyses were therefore performed to generate pooled point estimates and 95 % confidence intervals for the sensitivity and specificity of the biomarker under investigation with histopathological confirmation of acute appendicitis, together with hierarchical summary receiver operating characteristic (ROC) curves. The software used for this analysis was the custom-designed statistical package MIDAS [8]. Areas under the hierarchical summary ROC curves, and *I* ^2^ statistics, were obtained directly from the MIDAS output. See Zhou and Tu [9] for an in-depth description of the statistical methods used.

### Cost–benefit trade-off analysis

To evaluate the biomarkers, we applied decision analysis methodology, employing multi-criteria decision analysis (MCDA) [10] to assess trade-offs between cost (both time and financial) and benefit amongst the biomarkers, in terms of their performance characteristics (Table 2). The list of performance characteristics was grouped into three areas, namely monetary costs, time to results and benefits, encompassing all the remaining characteristics that were neither costs nor time. Through the literature review and expert survey, we determined the mean level of performance of the biomarkers on each of the characteristics. Criteria on which all biomarkers had identical performance, such as patient acceptability, were removed. The performance level was converted to a score by assigning a value of 0 to represent the worst performance (e.g., the highest unit price or worst sensitivity) and a value of 100 to represent the highest performance (e.g., lowest unit price or highest sensitivity). We assumed linearity between performance and value, such that for any intermediate level the corresponding value was interpolated from the worst and best performances on that criterion (valued 0 and 100, respectively).

**Table 2:** Performance of various biomarkers with respect to the surgeon rankings

Biomarker	Sens. (%)	Spec. (%)	Ease of test	Predictive of perforation (%)	Cost (£)	Time for result (h)	Acceptability	Reproducibility
WCC	79	55	Easy	69	2.5	1	Good	92
CRP	76	50	Easy	78	30	1	Good	81
Bilirubin	51	78	Easy	71	2	1	Good	98
Pro-calcitonin	36	88	Easy	83	17.42	12	Good	96
IL-6	73	72	Easy	84	15.5	168	Good	91
5-HIAA	72	86	Easy	0	21	240	Good	93
Surgeon rank	1	2	3	4	5	6	7	8

Acceptability considered ‘good’ as all can be done routinely. Ease of testing all considered ‘easy’ as all are noninvasive

*WCC* White cell count, *CRP* C-reactive protein, *IL*-*6* Interleukin 6, *5*-*HIAA* Urinary serotonin, *Sens* Sensitivity, *Spec* Specificity

Criteria weightings were derived from the rankings assigned by the European surgeons. The highest ranked criterion was given a weighting of 100, the second highest ranked criterion was given a weighting of 90, and so forth. The weightings were normalized so that they totaled 1, for each performance area. We applied a weighted average rule to combine the value scores across criteria as in:

$${\text{Value}} = \sum\limits_{k} {W_{k} {\text{Value}}_{k} } ,$$

where *V*_*k*_ indicates the value of an option on the *k*th criterion and *W*_*k*_ is the weighting assigned to that criterion. The overall value was therefore bounded between 0 and 100: A biomarker that had the worst performance on all the criteria would have an overall value of 0, whereas the biomarker that had the best performance on all the criteria would have an overall value of 100. The more desirable the biomarker was, the higher this value was. Two-way cost–benefit maps highlighted the trade-offs between different aspects of the biomarkers. Sensitivity analyses examined the robustness of the results. Trade-off analyses were performed using decision analytic software *HiView* (version 3.2.0.7, educational copy).

## Results

### Literature search

**Fig. 1:**
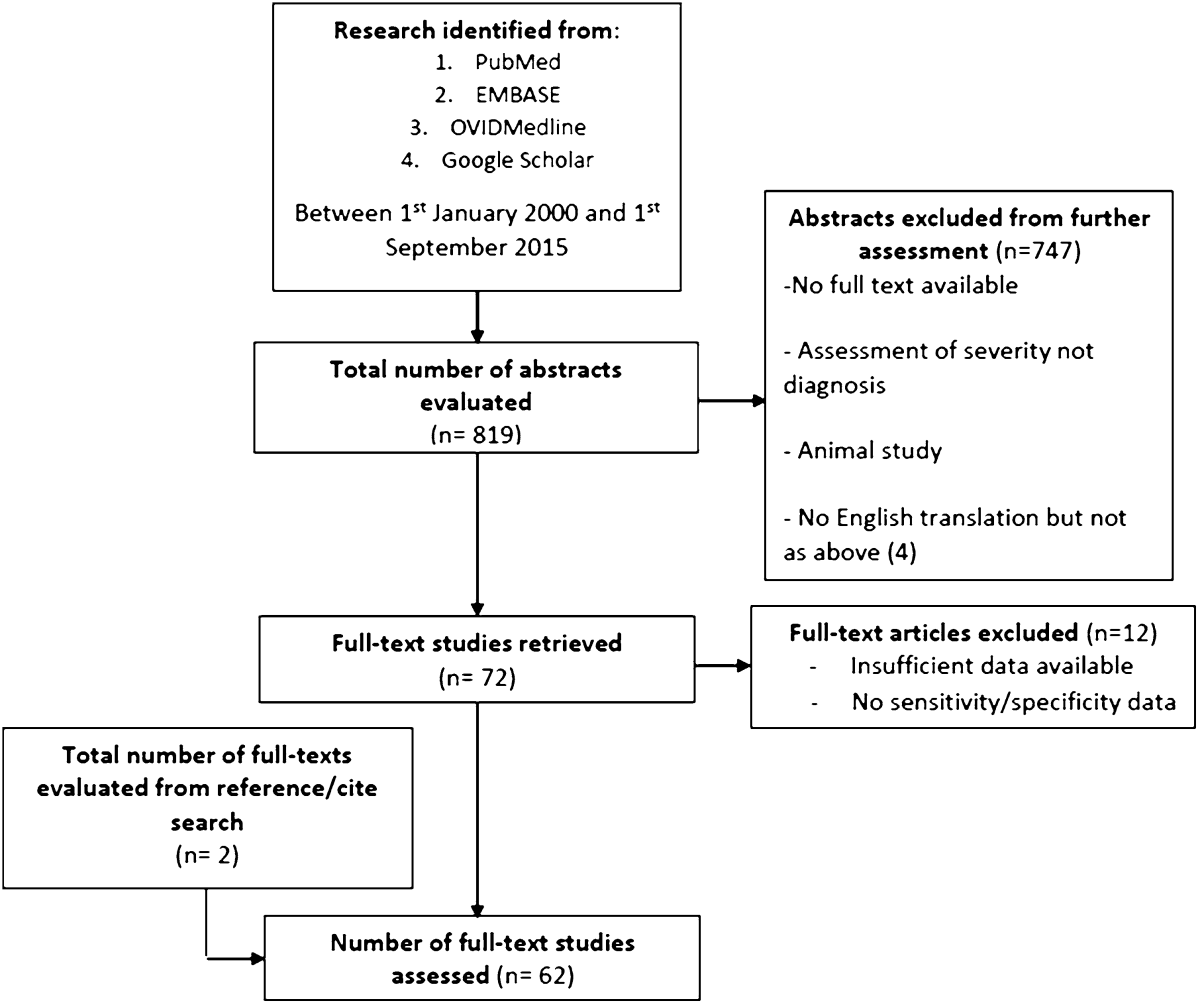
Schematic to show the strategic literature search

Sixty-two full-text articles met the inclusion criteria and were appraised following the literature search (Fig. 1). Forty-nine of these were used to assess the diagnostic accuracy of biomarkers. Eight studies assessed urinary markers (7 for urinary serotonin and 1 for leucine-rich glycoprotein). Forty-three studies investigated serum biomarkers (23 on white cell count, 24 on C-reactive protein, 13 on bilirubin, 3 on serum amyloid A, 1 on S100 A8/9 protein, 2 on calprotectin, 7 on pro-calcitonin, 1 on D-dimer, 5 on interleukin 6, 1 on interleukin 10, 1 on leucine-rich glycoprotein, 1 on fibrinogen, 1 on liposaccharide binding protein and 1 on high mobility group box protein-1). Thirty-seven studies assessed whether biomarkers were predictive of severity (20 on white cell count, 19 on C-reactive peptide, 19 on bilirubin, 7 on pro-calcitonin, 3 on interleukin 6 and 1 on urinary serotonin) [12, 15, 20, 21, 23, 24, 26–29, 31–33, 38–41, 43, 44, 47, 48, 50, 58, 60–72]. The demographics of these studies are shown in Appendixes 1 and 2 in ESM.

### QUADAS-2 evaluation

**Fig. 2:**
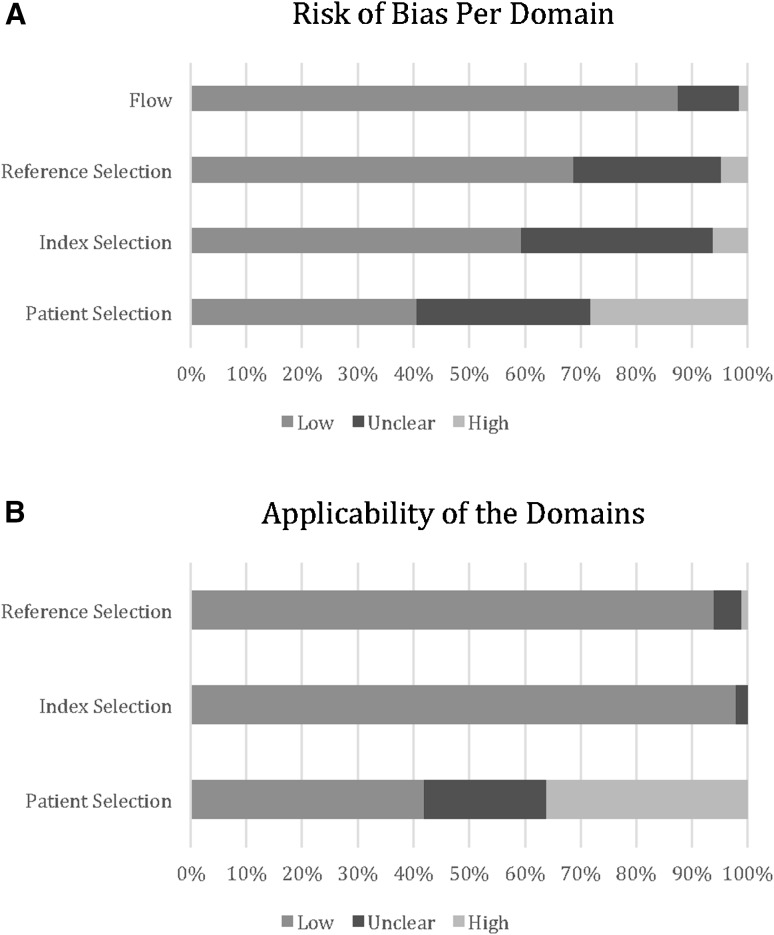
**A** Graph displaying the percentage of studies with varying degree of bias for each of the four QUADAS-2 domains. **B** Graph displaying the percentage of studies of varying applicability with respect to three of the four QUADAS-2 domains

The results of the QUADAS-2 assessment of the studies are shown in Fig. 2. Fifty-nine percent of studies had an ‘unclear’ or ‘high’ risk of bias with respect to patient selection due to constraining exclusion criteria. This limited the applicability of fifty-eight percent of the studies with respect to patient selection. Forty-one percent and thirty-one percent of studies had an ‘unclear’ or ‘high’ risk of bias with respect to the index and reference standards, respectively. This was due to a lack of information regarding blinding, thresholds and the order in which they were assessed. Only thirteen percent of studies had an ‘unclear’ or ‘high’ risk of bias with respect to the patient flow.

Biomarkers that were included in more than 2 studies were taken forward for pooled analysis.

### Pooled analysis for individual serum biomarkers in acute appendicitis

#### White cell count

The pooled sensitivity of white cell count for the diagnosis of acute appendicitis was 0.79 (95 % CI 0.78–0.81; *I* ^2^ = 92.0 %), and its pooled specificity was 0.55 (95 % CI 0.54–0.57; *I* ^2^ = 88.0 %). The area under the curve for the summary ROC was 0.75 ± 0.02. For the diagnosis of perforated appendicitis, the pooled sensitivity was 0.70 (95 % CI 0.68–0.73; *I* ^2^ = 95.5 %) and pooled specificity was 0.49 (95 % CI 0.48–0.50; *I* ^2^ = 98.5 %), giving an area under the curve of 0.69 ± 0.05.

#### C-reactive protein

The pooled sensitivity of C-reactive protein for the diagnosis of acute appendicitis was 0.76 (95 % CI 0.75–0.78; *I* ^2^ = 81.4 %), and its pooled specificity was 0.50 (95 % CI 0.48–0.52; *I* ^2^ = 94.2 %). The area under the curve for the summary ROC was 0.80 ± 0.02. For the diagnosis of perforated appendicitis, the pooled sensitivity was 0.76 (95 % CI 0.74–0.78; *I* ^2^ = 95.2 %) and pooled specificity was 0.52 (95 % CI 0.51–0.53; *I* ^2^ 98.4 %), giving an area under the curve of 0.78 ± 0.02.

#### Bilirubin

The pooled sensitivity of bilirubin for the diagnosis of acute appendicitis was 0.51 (95 % CI 0.50–0.52; *I* ^2^ = 97.7 %), and its pooled specificity was 0.78 (95 % CI 0.76–0.80; *I* ^2^ = 92.0 %). The area under the curve for the summary ROC was 0.72 ± 0.05. For the diagnosis of perforated appendicitis, the pooled sensitivity was 0.52 (95 % C.I 0.49–0.54; *I* ^2^ = 87.2 %) and pooled specificity was 0.76 (95 % CI 0.75–0.77; *I* ^2^ = 97.8 %), giving an area under the curve of 0.71 ± 0.04.

#### Pro-calcitonin

The pooled sensitivity of pro-calcitonin for the diagnosis of acute appendicitis was 0.36 (95 % CI 0.31–0.40; *I* ^2^ = 96.0 %), and its pooled specificity was 0.88 (95 % CI 0.83–0.91; *I* ^2^ = 81.8 %). The area under the curve for the summary ROC was 0.82 ± 0.10. For the diagnosis of perforated appendicitis, the pooled sensitivity was 0.69 (95 % CI 0.62–0.76; *I* ^2^ = 93 %) and pooled specificity was 0.67 (95 % CI 0.62–0.71; *I* ^2^ = 97 %), giving the area under the curve of 0.83 ± 0.07.

#### IL-6

The pooled sensitivity of IL-6 for the diagnosis of acute appendicitis was 0.73 (95 % CI 0.67–0.78; *I* ^2^ = 91.1 %), and its pooled specificity was 0.72 (95 % CI 0.63–0.79; *I* ^2^ = 62.3 %). The area under the curve for the summary ROC was 0.74 ± 0.04. For the diagnosis of perforated appendicitis, the pooled sensitivity was 0.79 (95 % CI 0.72–0.85; *I* ^2^ = 65.1 %) and pooled specificity was 0.62 (95 % CI 0.55–0.68; *I* ^2^ = 95 %), giving an area under the curve of 0.84 ± 0.03.

#### Pooled analysis for 5-HIAA from urine in acute appendicitis

The pooled sensitivity of urinary 5-HIAA for the diagnosis of acute appendicitis was 0.72 (95 % CI 0.68–0.76; *I* ^2^ = 93.4 %), and its pooled specificity was 0.86 (95 % CI 0.80–0.92; *I* ^2^ = 68 %). The area under the curve for the summary ROC was 0.88 ± 0.07. Pooled analysis for severity was precluded as only one study met the inclusion criteria.

#### Biomarker survey

Six hundred and eighty-eight surgeon members of the EAES responded to the survey (77 % of which were consultants, 18 % registrar level and 4 % other grades), giving a response rate of 12.7 %. Diagnostic sensitivity was given the highest average rank by the surgeon consensus and was thus weighted as the most important biomarker characteristic. The results of the other parameters are listed in Table 2.

#### Cost–benefit trade-off

Since all biomarkers had identical performances in terms of ‘ease of test’ and ‘acceptability,’ these two criteria were removed from the trade-off analysis. Table 3 displays the normalized weighted scores out of 100 for each of the six biomarkers with respect to the costs, time for result and benefits (diagnostic sensitivity, specificity, prediction of perforation and reproducibility), as well as an overall performance score.

**Table 3:** Normalized scores (out of 100) for the six biomarkers with respect to financial cost, time, diagnostic benefit (composite of sensitivity, specificity, reproducibility and prediction of perforation) and overall performance

	WCC	CRP	Bilirubin	Pro-calcitonin	IL-6	5-HIAA
Cost performance	98	0	100	45	52	32
Time performance	100	100	100	95	30	0
Diagnostic benefit	64.3	45	44	58	53	87
Overall performance	74.6	52.0	75.1	65.0	68.3	52.2

*WCC* White cell count, *CRP* C-reactive protein, *IL*-*6* Interleukin 6, *5*-*HIAA* Urinary serotonin

**Fig. 3:**
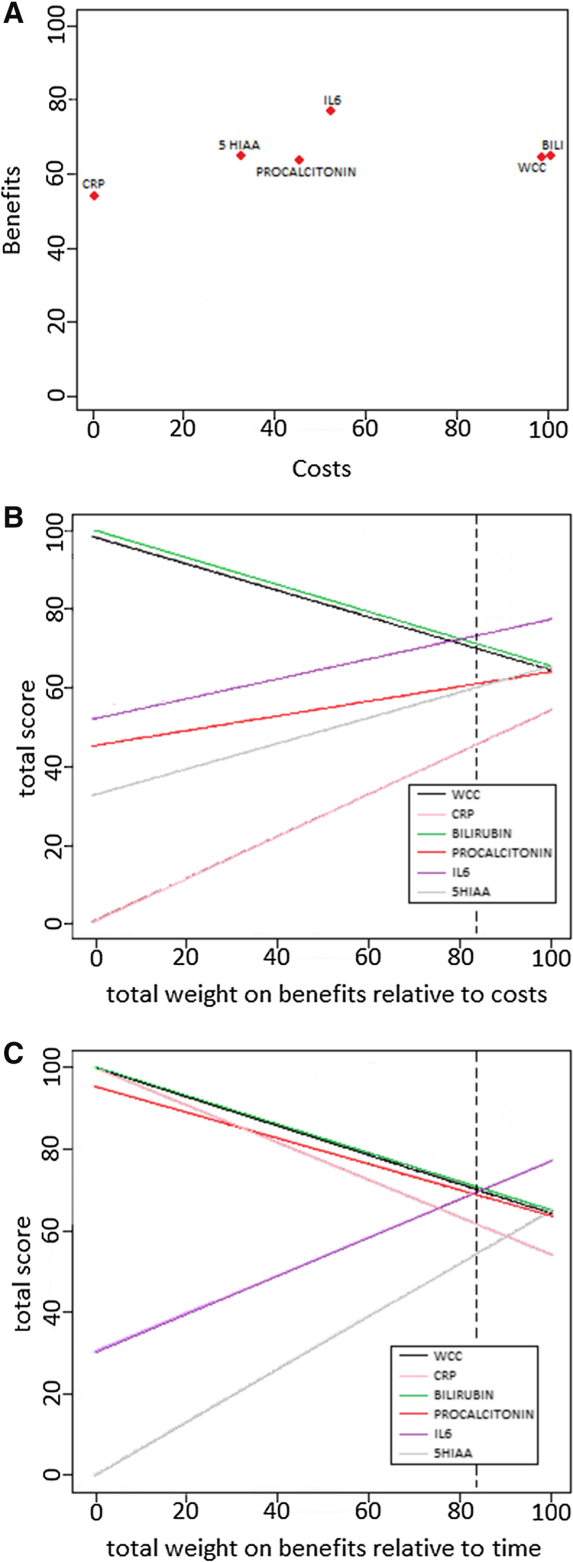
**A** Cost–benefit trade-off for the six biomarkers. Benefits include a summation sensitivity, specificity, predictive ability and reproducibility. **B** Sensitivity analysis revealing the effect of changing the current weighting (*dashed line*) placed upon financial cost and overall benefits. **C** Sensitivity analysis revealing the effect of changing the current weighting (*dashed line*) placed upon time for result and overall benefits

Figure 3A displays trade-offs between the benefits, as defined above, and the costs. White cell count and bilirubin performed best overall with the latter scoring marginally higher. When appraising the benefits in isolation, interleukin-6 performed the best. Sensitivity analysis demonstrated how the performance of the biomarkers would change if the relative importance of the various characteristics, as determined by the survey, was altered. If less importance was placed upon the financial cost or the time for result than its relative benefits (such as sensitivity), then the surgeons’ preference would be shifted further in favor of novel markers such as IL-6 (Fig. 3B, C).

The remaining biomarkers (C-reactive peptide, serotonin and pro-calcitonin) were inferior to those previously mentioned in a way that probabilistically dominated by the other three tests.

## Discussion

This study has highlighted the variable performance of biomarkers in the diagnosis of appendicitis, which reduces their potential to provide established objective criteria when used in isolation. This analysis has shown that whilst traditional markers including white cell count are associated with low temporal and financial cost, their overall diagnostic accuracy is relatively poor. As such weighting the analysis in favor of diagnostic characteristics such as high sensitivity or specificity, as opposed to process-related performance, would favor the use of novel biomarkers. The low diagnostic accuracy of elevated WCC is likely due to the presence of the underlying generalized inflammatory process seen with acute appendicitis, but also a number of other inflammatory conditions [12]. Conversely, novel markers that are less commonly used clinically in the diagnosis of appendicitis such as interleukin-6 have been shown to have a higher diagnostic benefit, but are associated with significant costs. The results of the literature search also highlight the expansion of work to look for novel diagnostic biomarkers, which to date remain only tested in isolated studies preventing meaningful analysis for clinical application [34].

There was a ‘high’ or ‘unclear’ risk of bias in 59 % of the studies with respect to patient selection. This was due to insufficient information regarding selection criteria. A number of studies assessing novel markers utilized healthy controls, or for example, with bilirubin, excluded patients in whom this could be caused by alternative pathology. This, however, leads to a selection bias when assessing the diagnostic ability of the biomarker with respect to suspected appendicitis and can spuriously improve the specificity. There was also an ‘unclear’ bias with respect to the index tests, especially with novel biomarkers, as diagnostic thresholds were not stated. The majority of the studies showed good applicability, but the assessment of a restricted demographic, such as pediatric patients, limited the studies performance with respect to this domain.

This study has highlighted the challenges associated with using single biomarkers in the diagnosis of appendicitis. Radiological investigation, especially CT, has been shown to have far superior diagnostic ability, with a reported sensitivity and specificity of 94 and 95 %, respectively [73]. However, the estimated radiation dose associated with a CT abdomen is 14mSV, equating to an increase of 0.2 % in the cancer risk for a 30-year-old patient [74]. Furthermore, CT remains a relatively expensive modality that could not be practically used in all patients in many areas of the world. Several studies have already suggested the use of diagnostic algorithms to ensure judicious use of radiology [73] and have demonstrated the potential to halve the use of CT scanning without increasing the negative appendectomy rate. Biomarkers could therefore be incorporated into these diagnostic algorithms in order to rationalize and more appropriately allocate further investigations.

Previous studies on biomarkers in appendicitis have focused solely upon their diagnostic accuracy. However, this study has highlighted the importance of considering clinical utility when assessing biomarkers. Interleukin-6 had the overall highest overall beneficial characteristics; however, this neglects its 168-h process time and expensive cost per test, which would preclude it from actual clinical use. This is further highlighted by the sensitivity analysis, which demonstrated that factoring in the significance of costs, more traditional biomarkers such as WCC, will be preferred. This study has therefore highlights the potential importance of cost–benefit modeling to improve this decision-making process when considering regional or national allocation of resources for diagnostic investigations.

In fact, no single biomarker had all the desired characteristics for the diagnosis of acute appendicitis. More commonly used biomarkers have less process-related costs due to the widespread availability of the testing, but are of relatively poor diagnostic accuracy when used in isolation. New proposed biomarkers whilst having high diagnostic value often require more complex assays, in which some circumstances require them to be sent to regional centers for analysis. However, a combination of biomarkers, as is used by some institutions clinically with white cell count and CRP, may improve the diagnostic ability [41, 45]. Alternatively, the use of a biomarker in conjunction with a consistent clinical history and examination may improve diagnostic accuracy in a more feasible manner. This could be achieved by utilization of stratification scores such as the Alvarado, which is a 10-point scoring system incorporating the typical signs and symptoms seen with appendicitis. With a cutoff of 7, this diagnostic algorithm has been shown to have a reported specificity as high as 100 % [75]. However, the limitation of the utilization of these scoring systems is the subjective interpretation of clinical history and examination findings [42]. Furthermore, a surgeon’s clinical impression has in some cases been shown to be of equivalent diagnostic value as these scoring systems, highlighting the value of clinical experience and the limitations of the widespread utilization of scoring systems [36]. In effect, therefore we have shown that clinically white cell count and bilirubin should be considered of greater use in the diagnosis of acute appendicitis when compared other biomarkers. However, given the limitations associated with current biomarkers, a high level of discrimination is required when interpreting these in practice, and the use of clinical impression in conjunction with radiological investigations remains the mainstay of the diagnostic paradigm.

The limitations of this study are primarily as a result of the studies included to inform the cost–benefit trade-off. Patient selection varied, and a lack of details regarding exclusion criteria limited the applicability of the studies to a patient population. Moreover, there was heterogeneity in the study designs, with a number of retrospective studies being included. Many of these trials did not explicitly mention blinding of the investigators, which is another potential source for bias and limitation of this review. Inherently with the use of novel biomarkers, no preexisting widely accepted threshold exists, leading many studies to assess various diagnostic cutoff values. Without blinding the investigators to the results of the histology, this increases the scope for bias. Furthermore, these studies often employed ‘healthy’ controls to formulate the testing thresholds; however, minimal details were provided as to the demographics of these controls, as well as leading to the aforementioned issues regarding specificity. A further limitation of this type of review is the potential for publication bias. Whilst this was mitigated by conducting a thorough multi-database search, the presence of language and publication bias still persists.

The results are further limited by the fact that the weighting was based upon the results of an online survey which had a response rate of 12.7 % and represented only surgeons affiliated with the EAES. Moreover, as the best overall marker changed with increasing the importance of sensitivity, the reliance upon the weighting system demonstrates how the conclusions would change depending on the opinions of the surgeons.

## Conclusion

Appendicitis continues to pose a diagnostic challenge to emergency physicians and surgeons. Clinical impression remains a crucial tool in diagnosis, and treatment allocation in those with suspected appendicitis. As yet no biomarker has been shown to have sufficient diagnostic performance to be used in isolation clinically. This would suggest that further areas of research should focus upon the search for new novel diagnostic tests and the clinical utility of the tests, rather than repeat existing research into previously studied biomarkers. Through this approach, the accuracy of diagnosis of appendicitis can be enhanced, reducing the number of negative appendectomies performed, implied adverse impact to patients and treatment costs to hospitals.

## Electronic supplementary material

Below is the link to the electronic supplementary material.

Supplementary material 1 (DOCX 22 kb)
