# Supplementary material for: Biomarkers of acute appendicitis: systematic review and cost–benefit trade-off analysis
Source: Surg Endosc. 2016 Aug 5;31(3):1022–31. doi: 10.1007/s00464-016-5109-1 (PMC5315733; doi:10.1007/s00464-016-5109-1)
Supplement: Supplementary file 1 — Supplementary material 1 (DOCX 22 kb) [file 464_2016_5109_MOESM1_ESM.docx]

**Appendix 1:** Sensitivities and specificities of serum biomarkers assessed by the studies. Study types *P:* Prospective and *R:* Retrospective. Biomarkers *WCC:* White cell count, *CRP:* C-reactive protein, *Bili:* Bilirubin, *PCT:* Pro-calcitonin, *Cal:* Calprotectin, *IL 6:* Interleukin 6, *IL 10:* Interleukin 10, *DD:* D Dimer, *HMBG:* High mobility group box protein-1, *LBP:* Liposacharide binding protein, *SAA:* Serum amyloid A, *LRG:* Leucine rich glycoprotein, *S100:* S100 A8/9 protein and *Fib:* Fibrinogen.

| **Study** | **Type** | **Number of patients (n)** | **Biomarkers Assessed** | **Sensitivity** | **Specificity** | **Reference** |
| --- | --- | --- | --- | --- | --- | --- |
| Abbas et al. 2014 | P | 147 | CRP | 0.75 | 0.72 |  |
|  |  |  | PCT | 0.85 | 0.74 | ^11^ |
|  |  |  | SAA | 0.92 | 0.72 |  |
| Al-Abed et al. 2014 | R | 447 | WCC | 0.74 | 0.56 |  |
|  |  |  | CRP | 0.78 | 0.58 |  |
|  |  |  | Bili | 0.46 | 0.80 | ^12^ |
| Albayrak et al. 2011 | P | 80 | HMBG | 0.72 | 0.75 | ^13^ |
| Asfar et al. 2000 | P | 78 | CRP | 0.87 | 0.93 | ^14^ |
| Atahan et al. 2011 | R | 351 | Bili | 0.80 | 0.84 | ^15^ |
| Bealer and Colgin 2010 | P | 181 | WCC | 0.63 | 0.67 | ^16^ |
| Beltrán et al. 2007 | P | 198 | S100 | 0.93 | 0.54 |  |
|  |  |  | WCC | 1.00 | 0.41 |  |
|  |  |  | CRP | 0.90 | 0.29 | ^17^ |
| Bolandparvaz et al. 2004 | P | 110 | WCC | 0.70 | 0.66 |  |
|  |  |  | CRP | 0.58 | 0.60 | ^18^ |
| Cardall et al. 2004 | P | 293 | WCC | 0.76 | 0.47 | ^19^ |
| Chandel et al. 2011 | P | 28 | PCT | 0.96 | 1.00 | ^20^ |
| Emmanuel et al. 2011 | R | 472 | Bili | 0.34 | 0.88 | ^21^ |
| Erkasap et al. 2000 | P | 102 | CRP | 0.92 | 0.87 | ^22^ |
|  |  |  | IL 6 | 0.33 | 0.83 |  |
| Estrada et al. 2007 | P | 170 | Bili | 0.56 | 0.69 | ^23^ |
| Farooqui et al. 2014 | R | 1008 | WCC | 0.68 | 0.64 |  |
|  |  |  | CRP | 0.32 | 0.81 | ^24^ |
|  |  |  | Bili | 0.69 | 0.56 |  |
| Groselj-Grenc et al. 2007 | P | 82 | WCC | 0.73 | 0.67 |  |
|  |  |  | CRP | 0.73 | 0.55 |  |
|  |  |  | IL 6 | 0.73 | 0.70 | ^25^ |
|  |  |  | LBP | 0.84 | 0.52 |  |
| Gurleyik et al. 2002 | P | 77 | IL 6 | 0.84 | 0.46 | ^26^ |
| Hong et al. 2012 | R | 1195 | Bili | 0.32 | 0.84 | ^27^ |
| Kaser et al. 2010 | R | 1073 | Bili | 0.38 | 0.78 | ^28^ |
| Kaya et al. 2012 | P | 78 | WCC | 0.85 | 0.25 |  |
|  |  |  | CRP | 0.72 | 0.75 |  |
|  |  |  | PCT | 0.24 | 0.50 | ^29^ |
|  |  |  | DD | 0.28 | 0.75 |  |
| Keskek et al. 2008 | R | 540 | WCC | 0.84 | 0.53 | ^30^ |
| Khan et al. 2004 | P | 259 | WCC | 0.83 | 0.38 |  |
|  |  |  | CRP | 0.76 | 0.84 | ^31^ |
| Khan 2008 | P | 110 | Bili | 0.82 | 1.00 | ^32^ |
| Khan 2009 | P | 122 | Bili | 0.70 | 0.25 | ^33^ |
| Kharbanda et al. 2012 | P | 176 | WCC | 1.00 | 0.42 |  |
|  |  |  | Cal | 1.00 | 0.27 | ^34^ |
|  |  |  | LRG | 1.00 | 0.35 |  |
| Kouame et al. 2005 | P | 70 | PCT | 0.50 | 0.88 | ^35^ |
| Laméris et al. 2009 | R | 942 | WCC | 0.81 | 0.43 |  |
|  |  |  | CRP | 0.79 | 0.31 | ^36^ |
| Lycopoulou et al. 2005 | P | 60 | WCC | 0.76 | 0.78 |  |
|  |  |  | CRP | 0.62 | 0.94 | ^37^ |
|  |  |  | SAA | 0.86 | 0.83 |  |
| McGowan et al. 2013 | R | 1271 | Bili | 0.55 | 0.90 | ^38^ |
| Mentes et al. 2012 | P | 201 | WCC | 0.64 | 0.77 | ^39^ |
|  |  |  | Fib | 0.63 | 0.50 |  |
| Paajanen et al. 2002 | P | 80 | WCC | 0.75 | 0.65 |  |
|  |  |  | CRP | 0.75 | 0.53 | ^40^ |
|  |  |  | IL 6 | 0.84 | 0.76 |  |
| Panagiotopoulou et al. 2013 | R | 1169 | WCC | 0.84 | 0.58 |  |
|  |  |  | CRP | 0.70 | 0.66 | ^41^ |
|  |  |  | Bili | 0.51 | 0.74 |  |
| Pruekprasert et al. 2004 | P | 193 | CRP | 0.62 | 0.53 | ^42^ |
| Sand et al. 2009 | P | 103 | WCC | 0.58 | 1.00 |  |
|  |  |  | CRP | 0.81 | 0.60 | ^43^ |
|  |  |  | PCT | 0.14 | 1.00 |  |
| Sand et al. 2009 | R | 538 | Bili | 0.70 | 0.86 | ^44^ |
| Schellekens et al. 2013 | P | 233 | WCC | 0.78 | 0.78 |  |
|  |  |  | CRP | 0.86 | 0.56 |  |
|  |  |  | SAA | 0.77 | 0.69 | ^45^ |
|  |  |  | Cal | 0.45 | 0.83 |  |
| Sengupta et al. 2009 | P | 98 | WCC | 0.85 | 0.72 |  |
|  |  |  | CRP | 0.65 | 0.68 | ^46^ |
| Souza et al. 2013 | P | 242 | WCC | 0.68 | 0.70 |  |
|  |  |  | CRP | 0.82 | 0.62 | ^47^ |
|  |  |  | Bili | 0.27 | 0.96 |  |
| Vaziri et al. 2014 | P | 100 | PCT | 0.43 | 1.00 | ^48^ |
| Wu et al. 2005 | R | 542 | CRP | 0.72 | 0.83 | ^49^ |
| Wu et al. 2012 | P | 214 | CRP | 0.85 | 0.27 | ^50^ |
|  |  |  | PCT | 0.96 | 0.17 |  |
| Xharra et al. 2012 | P | 173 | WCC | 0.85 | 0.68 | ^51^ |
|  |  |  | CRP | 0.85 | 0.72 |  |
| Yang et al. 2006 | R | 897 | WCC | 0.86 | 0.32 |  |
|  |  |  | CRP | 0.76 | 0.26 | ^52^ |
| Yildirim et al. 2006 | P | 85 | WCC | 0.87 | 0.64 |  |
|  |  |  | CRP | 0.70 | 0.64 |  |
|  |  |  | IL 6 | 0.79 | 0.50 | ^53^ |
|  |  |  | IL 10 | 0.41 | 0.93 |  |

**Appendix 2:** Sensitivities and specificities of urinary biomarkers assessed by the studies. Study type *P:* Prospective. Biomarkers, *5HIAA:* 5 Hydroxy-indolacetic acid, *LRG:* Leucine rich glycoprotein.

| **Study** | **Type** | **Number of patients (n)** | **Biomarkers Assessed** | **Sensitivity** | **Specificity** | **Reference** |
| --- | --- | --- | --- | --- | --- | --- |
| Bolandparvaz et al. 2004 | P | 110 | 5HIAA | 0.85 | 0.86 | ^18^ |
| Hernandez et al. 2008 | P | 72 | 5HIAA | 0.67 | 0.38 | ^54^ |
| Ilkhanizadeh et al. 2001 | P | 80 | 5HIAA | 0.98 | 1.00 | ^55^ |
| Jangjoo et al. 2012 | P | 70 | 5HIAA | 0.44 | 0.82 | ^56^ |
| Kharbanda et al. 2012 | P | 176 | LRG | 1.00 | 0.23 | ^34^ |
| Mihmanli et al. 2004 | P | 43 | 5HIAA | 0.23 | 0.95 | ^57^ |
| Oruc et al. 2004 | P | 36 | 5HIAA | 0.58 | 1.00 | ^58^ |
| Sarhan et al 2013 | P | 147 | 5HIAA | 0.81 | 0.85 | ^59^ |
